# Curcumin- and resveratrol-co-loaded nanoparticles in synergistic treatment of hepatocellular carcinoma

**DOI:** 10.1186/s12951-022-01554-y

**Published:** 2022-07-20

**Authors:** Yongshun Zheng, Ran Jia, Jun Li, Xiaohe Tian, Yeben Qian

**Affiliations:** 1 Department of General Surgery, The First Affiliated Hospital of Anhui Medical University, 218 Jixi Road, Hefei, 230022 Anhui China; 2 Department of Radiology and National Clinical Research Center for Geriatrics, Functional and Molecular Imaging Key Laboratory of Sichuan Province, Huaxi MR Research Centre (HMRRC), West China Hospital of Sichuan University, Chengdu, 610000 China; 3 Department of Chemistry, Key Laboratory of Functional Inorganic Material Chemistry of Anhui Province, School of Life Science, Anhui University, Hefei, 230000 China

**Keywords:** Nanoparticles, Curcumin, Resveratrol, Tumour-specific targeting, HCC, Chemotherapy

## Abstract

**Background:**

Currently, systemic therapies for patients with advanced-stage hepatocellular carcinoma (HCC) rely mainly on systemic drugs. However, traditional systemic drugs have a high rate of serious adverse events, and the curative effects of some potential anticancer drugs, such as curcumin (CUR) and resveratrol (RSV), are less apparent due to their poor bioavailability. Therefore, it is urgent to develop a highly effective therapy to improve patient prognosis. Herein, an injectable HCC-targeted nanoparticle (NP) was designed to deliver CUR and RSV to hepatoma cells.

**Results:**

The molecular self-assembled NPs showed higher tumour retention through the enhanced permeability and retention (EPR) effect of the NPs and surface modification with the HCC-specific peptide moiety SP94 to effectively treat HCC. These HCC-targeted NPs led to a significant reduction in the drug dosage, delayed the rate of drug release and improved the bioavailability of the encapsulated drugs. The drug concentrations in the vicinity of the tumour increased, and a good therapeutic effect was observed without obvious side effects.

**Conclusions:**

These SP94-mediated NPs allowed large amounts of antitumor drugs to accumulate in tumours, providing a novel strategy for innovative HCC therapy. This nanoplatform also offers an idea for exploring other potential chemotherapeutics.

**Graphical Abstract:**

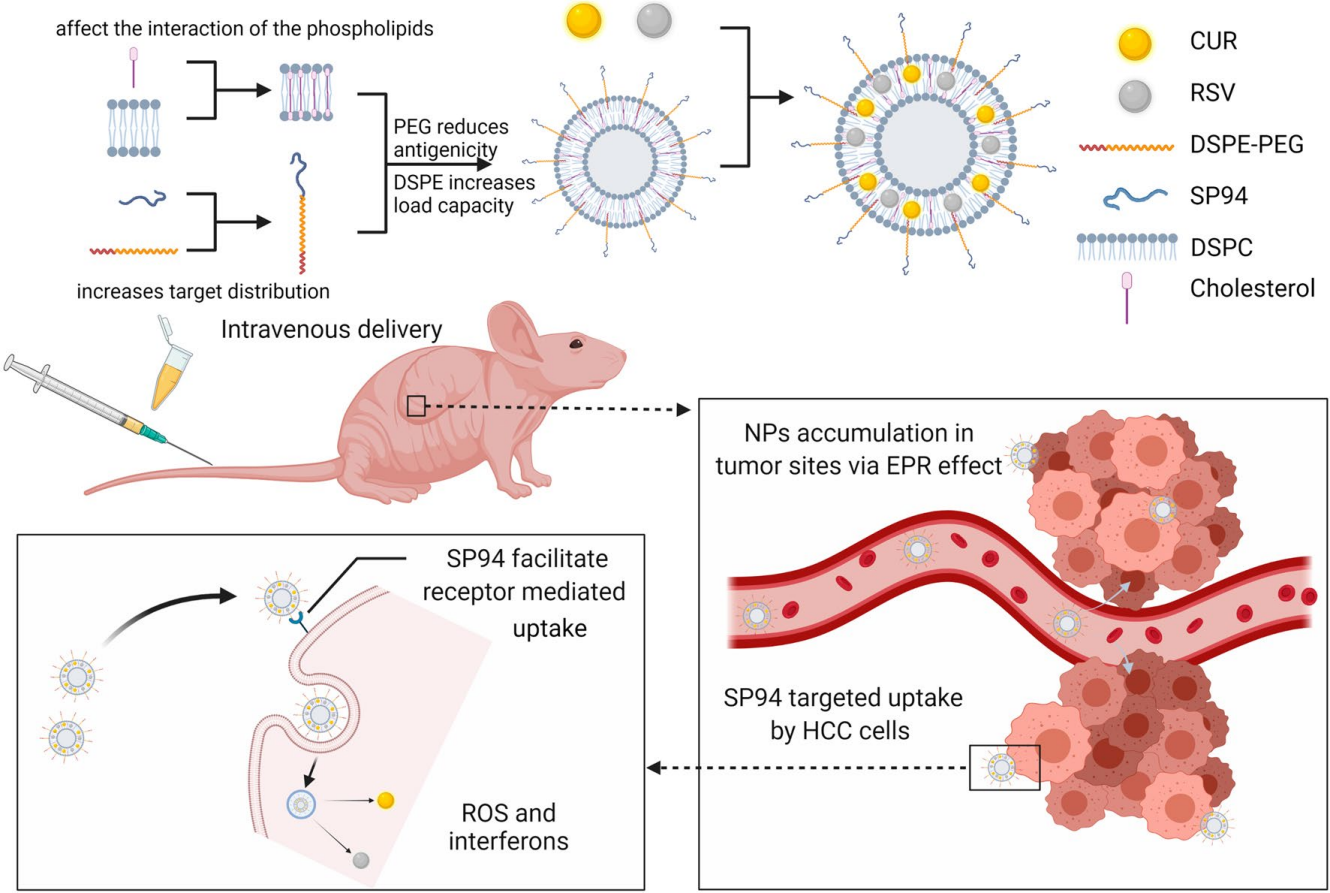

**Supplementary Information:**

The online version contains supplementary material available at 10.1186/s12951-022-01554-y.

## Background

As the sixth most common type of tumour and the fourth leading cause of cancer-related deaths worldwide, hepatocellular carcinoma (HCC) has become a severe medical problem [1]. Due to the lack of specific symptoms in the early stage of disease, most HCC patients are not diagnosed until they reach the advanced stage [2, 3]. Currently, the overall median survival time of patients suffering from intermediate and advanced HCC is less than 6 months. Although surgery is one of the best options for HCC treatments, patients with advanced HCC are often not candidates for surgery, and systemic therapy remains as the only option for palliative care [4]. However, the standard of care for frontline therapy with sorafenib has limited effects, and patients show clear drug resistance and poor tolerance [5]. Many patients discontinue treatment due to significant side effects, including hypertension, decreased weight, and palmar–plantar erythrodysesthesia [6]. Insufficient antitumor activity, liver toxicity in the context of cirrhosis, and inappropriate patient selection have been considered the reasons for treatment failure, so the abovementioned treatments remain unsatisfactory [3]. Therefore, there is an urgent need to develop novel therapies to treat this serious disease.

The beneficial effects of dietary curcumin (CUR) and resveratrol (RSV) on human health have attracted considerable research attention [7]. As a diketone compound, CUR is mainly derived from turmeric and has been used for centuries for its medicinal properties. Due to its unique aroma and colour, CUR is also widely used as a flavouring and colouring agent in the food industry [8]. Additionally, its intrinsic fluorescence property has been used to monitor intracellular uptake profiles [9], while its biological activities, such as antioxidant, apoptotic and anti-inflammatory effects, have been utilized for the treatment of a variety of diseases. Recent studies have focused on the functions of CUR in cancer suppression, including in gastric cancer [10], cervical cancer [11], HCC [12] and so forth. For instance, it has been demonstrated that CUR can cause DNA damage and arrest the cell cycle at G2/M phases in HeLa cells [11]. Additionally, CUR can inhibit the proliferation of various liver cancer cell lines and induce HepG2 cell apoptosis by enhancing reactive oxygen species (ROS) generation and regulating the TGF-β1/Smad3 signalling pathway [12, 13]. Similarly, RSV is a natural antioxidant polyphenolic compound that is found in a variety of plants, such as those that produce grapes, peanuts, and berries [14]. Recent studies have suggested that RSV can prevent or inhibit the development of breast [15], colorectal [16], pancreatic [17], prostate [18], and liver cancers [19]. For example, RSV inhibits breast cancer cell proliferation by regulating the STAT3 signalling cascade via its antioxidant and anti-inflammatory effects [20]. Additionally, RSV has been shown to induce malignant hepatic tissue apoptosis by activating caspase-3, upregulating the ratio of Bax/Bcl-2, and increasing p53 expression [21]. It is also worth noting that the administration of either CUR or RSV in combination with cisplatin has been used for treating various cancers, which reduces the chemoresistance and side effects of cisplatin [22–24]. The combination of CUR and RSV significantly sensitizes epithelial ovarian cancer cells to cisplatin [25], reduces doxorubicin-induced cardiotoxicity and effectively treats prostate cancer [26, 27]. CUR and RSV synergistically exert cytotoxic effects by inducing ER stress, increasing ROS generation, and activating autophagy and the PI3K/AKT/mTOR pathway [25, 28]. Since the cardio-, hepato-, nephro- and neuro-protective properties of CUR and RSV offer promising results for chemoprevention and chemoprotection in tumours, the potential synergistic effect of these drugs makes combination therapy an important research direction because it can further reduce the needed dosages and improve treatment potential [29]. However, there are currently no studies on CUR combined with RSV for the treatment of HCC, possibly because these drugs have low water solubility, poor chemical stability, and poor bioavailability, thus limiting their clinical application [8].

The abovementioned problems can be solved by nanotechnology-driven drug delivery systems (DDSs). 1,2-Distearoyl-sn-glycero-3-phosphoethanolamine-N-[maleimide (polyethylene glycol)-2000] (DSPE-PEG_2000_-Mal), which has been approved by the Food and Drug Administration (FDA), is a conjugated polymer widely used in DDSs that contains hydrophilic PEG and hydrophobic DSPE. It can be used as an auxiliary material to functionalize various DDSs, including phospholipid-based nanoparticles (NPs) and inorganic material-based NPs [30]. PEG prolongs the blood circulation time of DDSs by reducing liposome clearance by the reticuloendothelial system [31], and DSPE is used as the hydrophobic structure of NPs for efficient drug loading [32]. Cholesterol plays an important role in maintaining liposome structure by affecting membrane permeability, fluidity and interactions between phospholipids [33]. Nanospheres are formed by the self-assembly of these amphiphilic polymers, and the drug is loaded into the inner hydrophobic core to increase its water solubility, stability, and half-life in the bloodstream [34]. CUR and RSV preferentially localize to the hydrophobic acyl chain region and are inserted on top of the hydroxyl group of the cholesterol, which can stiffen the liposome membrane [35, 36]. Therefore, the coexistence of CUR and RSV can synergistically stabilize liposomes, thereby enhancing delivery performance. Encapsulating these therapeutic drugs in NPs for cancer treatment could improve drug accumulation in tumours via the enhanced permeability and retention (EPR) effect, so this approach has received extensive attention [37]. To further improve delivery performance, the NP surface can be modified for better tumour retention. Common methods include the use of the transferrin receptor [38], functionalized magnetic nanocomposites [39], thermosensitive hydrogels [40], cyclodextrin nanosponges [41], and photosensitizers [42]. The short peptide ligand SP94 (sequence: SFSIIHTPILPL), which can selectively bind to various types of HCC cells, was discovered through an in vivo phase display technique [43]. Compared with normal cells, including normal hepatocytes, endothelial cells, and immune cells, SP94-modified NPs showed higher affinity for various types of hepatoma cells [44]. In addition, peptide ligands have the advantages of easy synthesis, low expense, and lower immunogenicity. Therefore, SP94-modified DDSs are a promising approach [45].

**Fig. 1:**
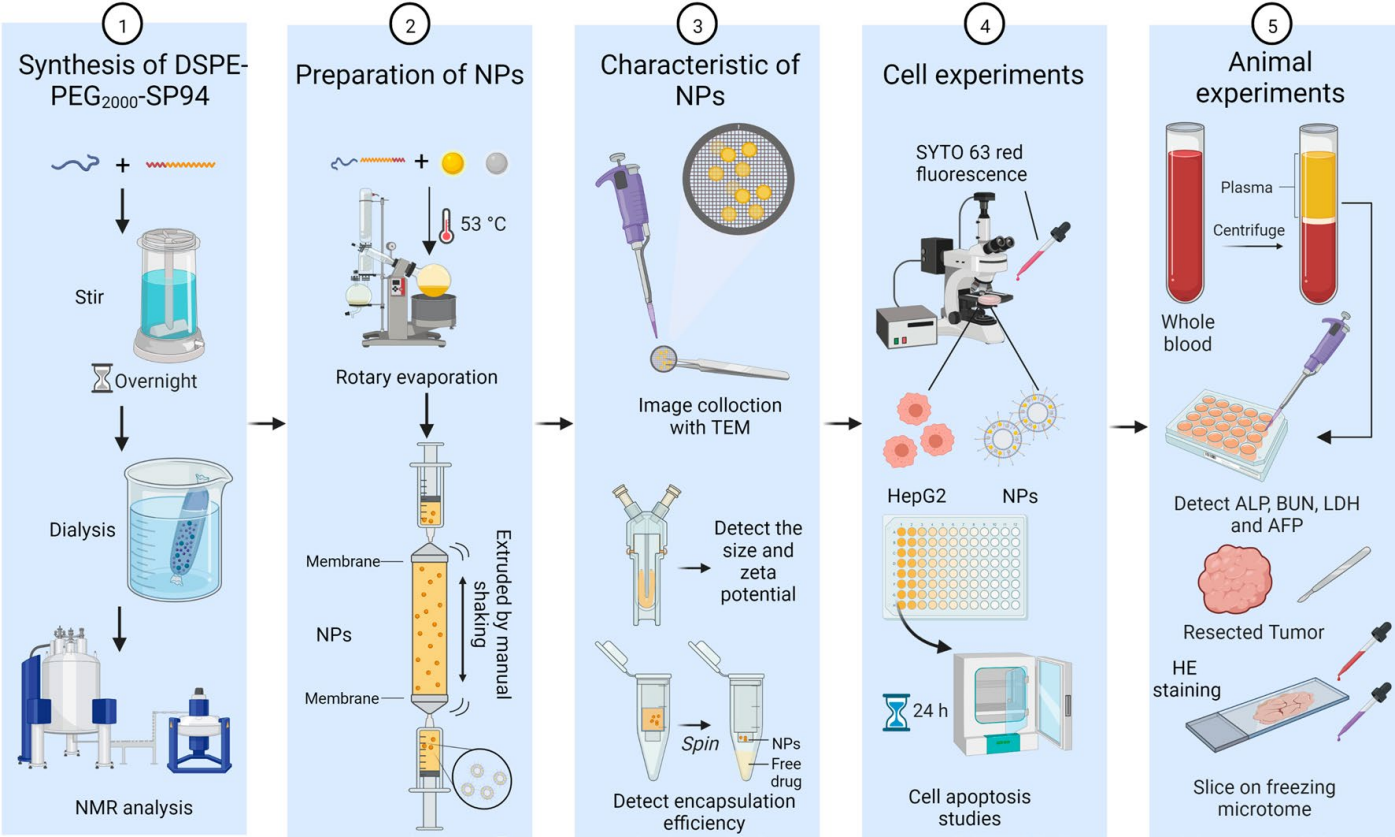
Schematic figure of depicting major materials and methods in current study

In this work, the rational design of HCC-targeted NPs loaded with CUR and RSV and their therapeutic effects on HCC cells and in animal models are presented. By coassembling RSV and CUR with amphiphilic lipids, cholesterol, and DSPE-PEG_2000_, a systemically injectable NP drug was formed. To further enhance the tumour-targeting ability, the NP surface was decorated with SP94 via a maleimide-thiol reaction. This kind of NP possesses the characteristics of higher drug loading, sustained drug release and colloidal stability. Cell experiments showed that the increased solubility of the drugs allows for easier cell uptake. On the other hand, animal experiments demonstrated that the sustained release of the drugs from the NPs avoids their rapid metabolism. This not only significantly lowers the drug dosage needed but also reduces the liver and kidney toxicity caused by the metabolized drugs. Most importantly, the dual effects of the EPR effect and SP94 peptide induction further increased the possibility of NP clustering near the tumour, thereby increasing the drug concentration in the tumour area and increasing the probability of tumour cell uptake. It has therefore been speculated that these targeted NPs offer more possibilities for the treatment of HCC by exploiting other potential anticancer drugs [46]. Major materials and methods in current study are shown in Fig. 1.

## Results and discussion

### Synthesis and characterization of DSPE-PEG_2000_-SP94 loaded with CUR and RSV

**Fig. 2:**
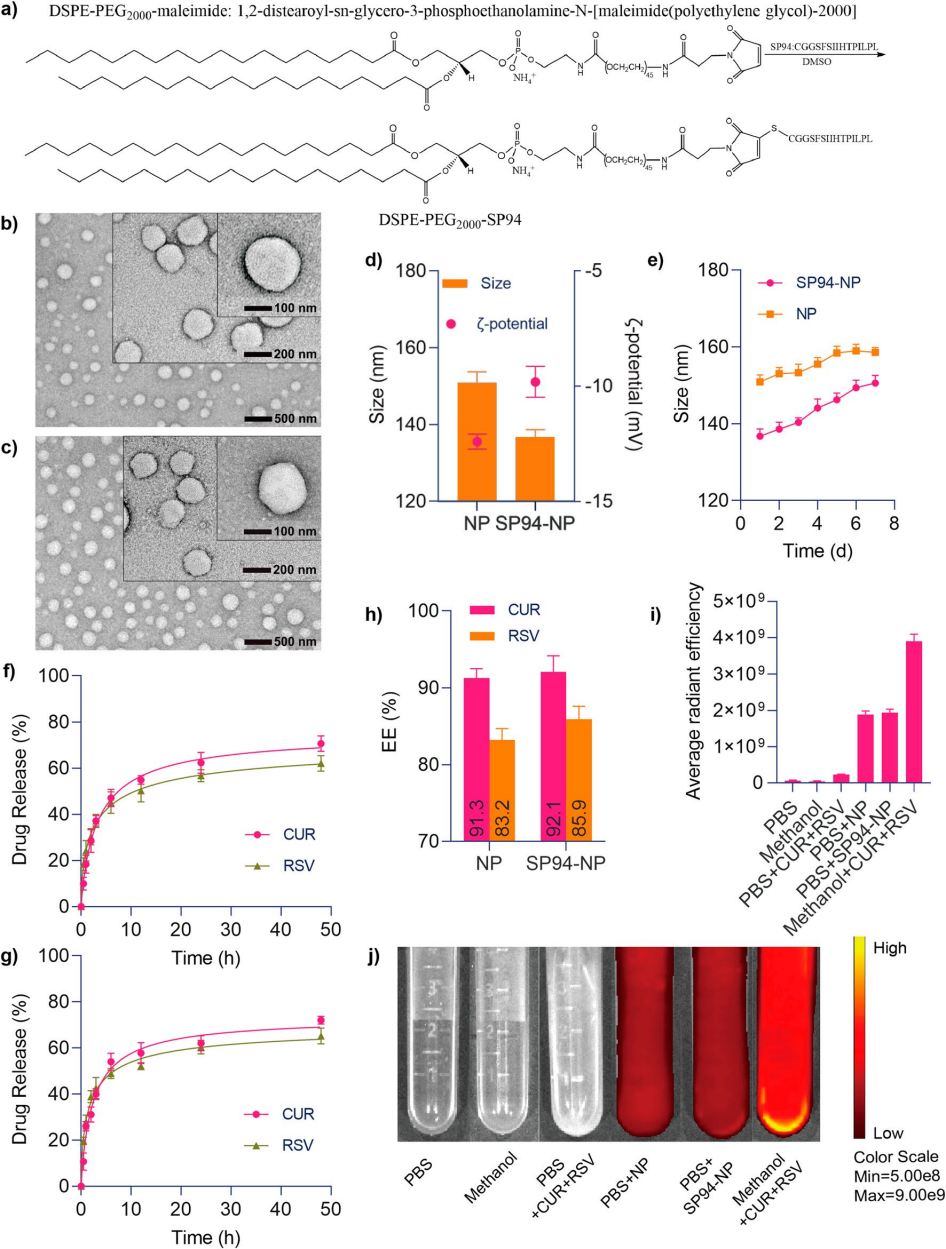
Characterization of CUR and RSV coloaded nanoparticles. **a** Synthesis of DSPE-PEG_2000_-SP94. Morphological images acquired by transmission electron microscopy (**b** NP, **c** SP94-NP). **d** Particle sizes and zeta potentials measured by dynamic light scattering. **e** Changes in the diameters of the NPs over the course of 7 days, indicating their stability. In vitro drug release profiles of CUR and RSV from the NPs determined by dialysis against PBS (pH 7.4) at 37 °C over 48 h (**f** NP, **g** SP94-NP). **h** NPs encapsulation efficiency of CUR and RSV. **i**, **j** The changes in CUR and RSV solubility in various solutions detected by a small animal imaging instrument and measurements of their average radiant efficiencies

The detailed methods for the preparation and characterization of DSPE-PEG_2000_-SP94 are described in the “Methods” section. In short, to overcome the low bioavailability of CUR and RSV and increase their anticancer efficacy, NPs were used for encapsulation. Amphiphilic DSPE-PEG_2000_ is easily miscible with various hydrophobic drugs and therefore widely used as a synthetic drug delivery matrix to protect the carrier from phagocytosis, thereby prolonging the blood circulation of the drugs. To further increase the targeting ability of the NPs, the SP94 peptide ligand was attached (the mass spectrometry and high-performance liquid chromatography (HPLC) analyses of SP94 are shown in Additional file 1: Figs. S1 and S2). SP94 was originally identified by the phage display technique, and it shows affinity for an unidentified receptor expressed on HCC cells [43]. NP surface modification with the SP94 ligand was achieved by coupling the thiolated peptide with maleimide-terminated DSPE-PEG_2000_ via a thiol-maleimide coupling reaction to form a covalent adduct (referred to as DSPE-PEG_2000_-SP94, Fig. 2a), which was verified by HPLC analysis (Additional file 1: Fig. S3) and 1H NMR spectroscopy (Additional file 1: Fig. S4). CUR + RSV and DSPE-PEG_2000_-SP94 constructed the targeted NPs by nanoprecipitation. Similarly, combining CUR + RSV and DSPE-PEG_2000_ formed nontargeted drugs. NP-CUR + RSV and SP94-NP-CUR + RSV referred to as NP and SP94-NP in figures.

The fundamental properties of the NPs, such as electron microscopy analyses of their morphology under different magnifications and particle sizes and zeta potentials, are presented in Fig. 2b–d, respectively. Previous studies have proven that the tumour vascular endothelium has a larger barrier gap than normal vascular endothelium, so the EPR effect significantly impacts the penetration of NPs into solid tumours, especially NPs below 200 nm [47]. Therefore, our NPs had a suitable size to aggregate at tumour sites. The long-term stability of the NPs was evaluated by storing the NP and SP94-NP solutions at room temperature for one week, and both NPs displayed little change in particle size (Fig. 2e). These results indicated that the two NPs are monodisperse with well-defined spherical nanostructures that show good stability and long-term storage capability.

### Kinetics of CUR and RSV release from the drug-loaded NPs

Next, the encapsulation efficiency (EE), drug release kinetics and solubility changes were evaluated. The amounts of CUR and RSV released from the NPs by dialysis against phosphate-buffered saline (PBS; pH 7.4) at 37 °C were determined by UV spectrophotometry (Fig. 2f, g). Both drugs showed relatively stable and slow release rates. After 48 h, the release rates tended to slow down, and the total amounts released were approximately 62.05–72.23%. Although the EEs of CUR and RSV were both greater than 80%, the EE for CUR was slightly larger than that of RSV, which suggested that this nanosystem has a better carrying capacity for CUR than RSV (Fig. 2h). CUR and RSV have low solubility in water but are completely soluble in methanol. A small animal imaging system was used to evaluate the change in drug solubility in water after nanomaterial encapsulation, and it was found that the nanosystem significantly enhanced the solubility of the drugs (Fig. 2i, j). These data clearly indicated that the NPs had a strong drug loading capacity and sustained drug release properties, which can help to deliver anticancer drugs to the tumour area.

### Intracellular assessment of the cellular uptake of SP94-NP

**Fig. 3:**
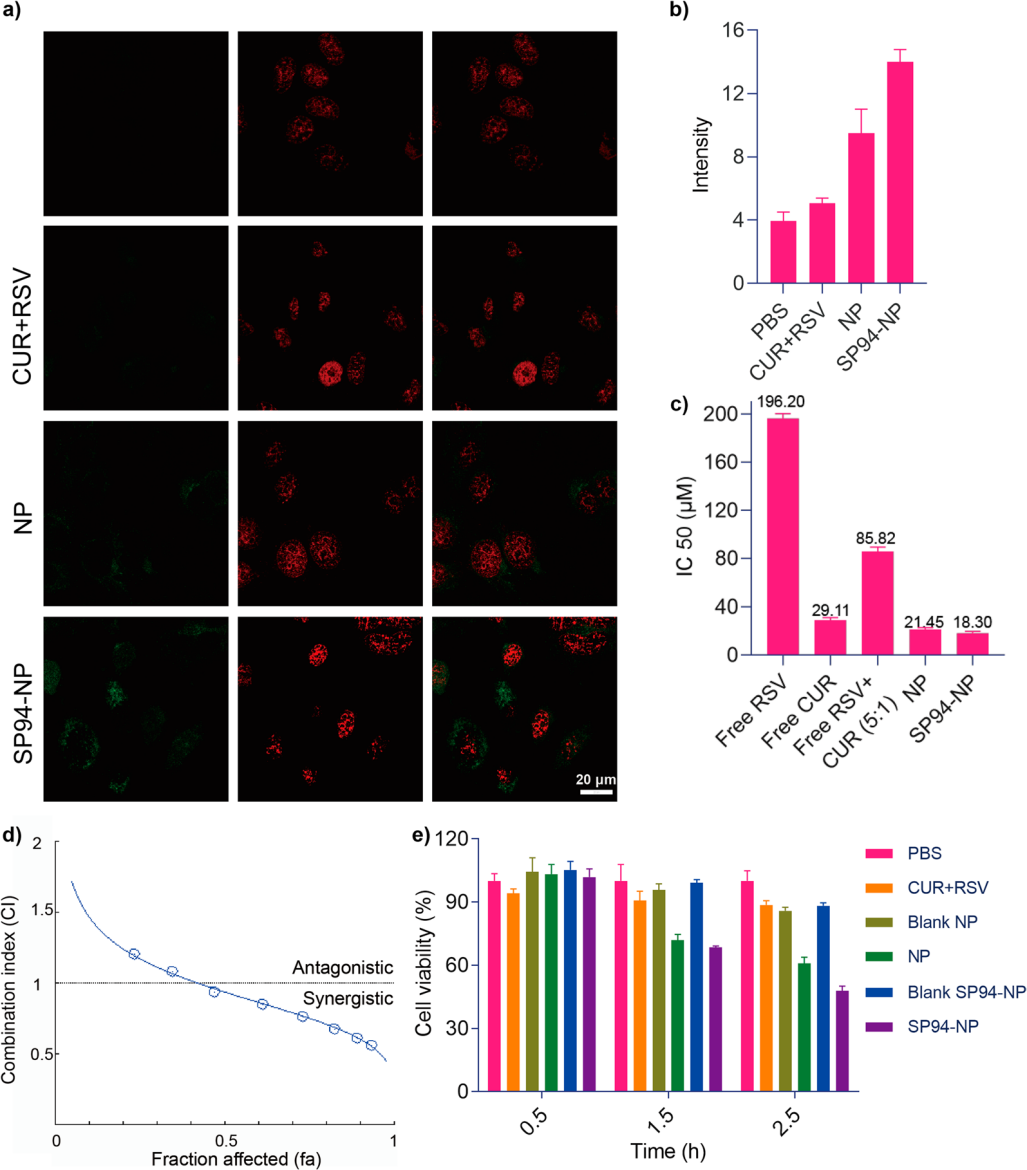
Cellular uptake of the NPs and the synergism of CUR and RSV. **a**, **b** Cellular uptake of the NPs was detected by a fluorescence survey of CUR and SYTO 63 with an inverted-type laser scanning confocal microscope. **c** IC_50_ values were used to determine the cytotoxicity of individual drug treatments. **d** CI values for CUR and RSV in HepG2 cells, with CI values of less than 1, 1, and more than 1 indicating synergistic, additive, and antagonistic effects, respectively, as calculated by CompuSyn software. **e** Effect of NPs on HepG2 cell viability at 0.5, 1.5, and 2.5 h

The uptake of the drug-loaded NPs by HepG2 cells was assessed using laser scanning confocal microscopy (LSCM). After treatment with PBS, CUR + RSV, NP and SP94-NP and SYTO 63, strong red fluorescence from SYTO 63 was observed. However, in NP-treated cells, the fluorescence signal was punctate in the cytoplasm and negligible in the nucleus (Fig. 3a, b). This distribution is typical, as NPs are internalized by cells via the endocytosis/lysosomal pathway. These results suggested that the drugs remain encapsulated by the NPs during internalization. Moreover, in the two HCC cell lines, the targeted SP94-NPs showed superior drug accumulation compared with the nontargeted NPs. Therefore, SP94-NP more effectively targets HCC tumour cells, which allows the drugs to accumulate effectively. Ultimately, this active targeting effect can reduce the in vivo toxicity of anticancer drugs and allow cancer cells to undergo apoptosis.

### Synergistic effects of CUR and RSV and cell viability in vitro

To elucidate whether the codelivery of CUR and RSV more effectively causes HepG2 cell apoptosis and whether the SP94-NPs can target cancer cells more effectively, the IC_50_ values of RSV, CUR, CUR + RSV, NP and SP94-NP were identified. The combination indices (CIs) of the two combined RSV and CUR treatments are listed in Fig. 3d. When the fraction of cells affected (fa) < 0.5, the two displayed a synergistic effect. The results showed a sequential decrease (Fig. 3c), which illustrated that CUR and RSV had synergistic effects and that the SP94-NPs can achieve the same effect with a lower dosage.

Figure 3e shows that the blank NPs had almost no toxicity while the drug-loaded NPs had clear inhibitory effects on cell viability after 1.5 h, which demonstrated that the nanomaterial itself was safe, and the NPs, especially those modified with SP94, were more conducive to drug uptake by HepG2 cells.

### Evaluation of the therapeutic mechanism of the cytotoxicity against HCC cells in vitro

**Fig. 4:**
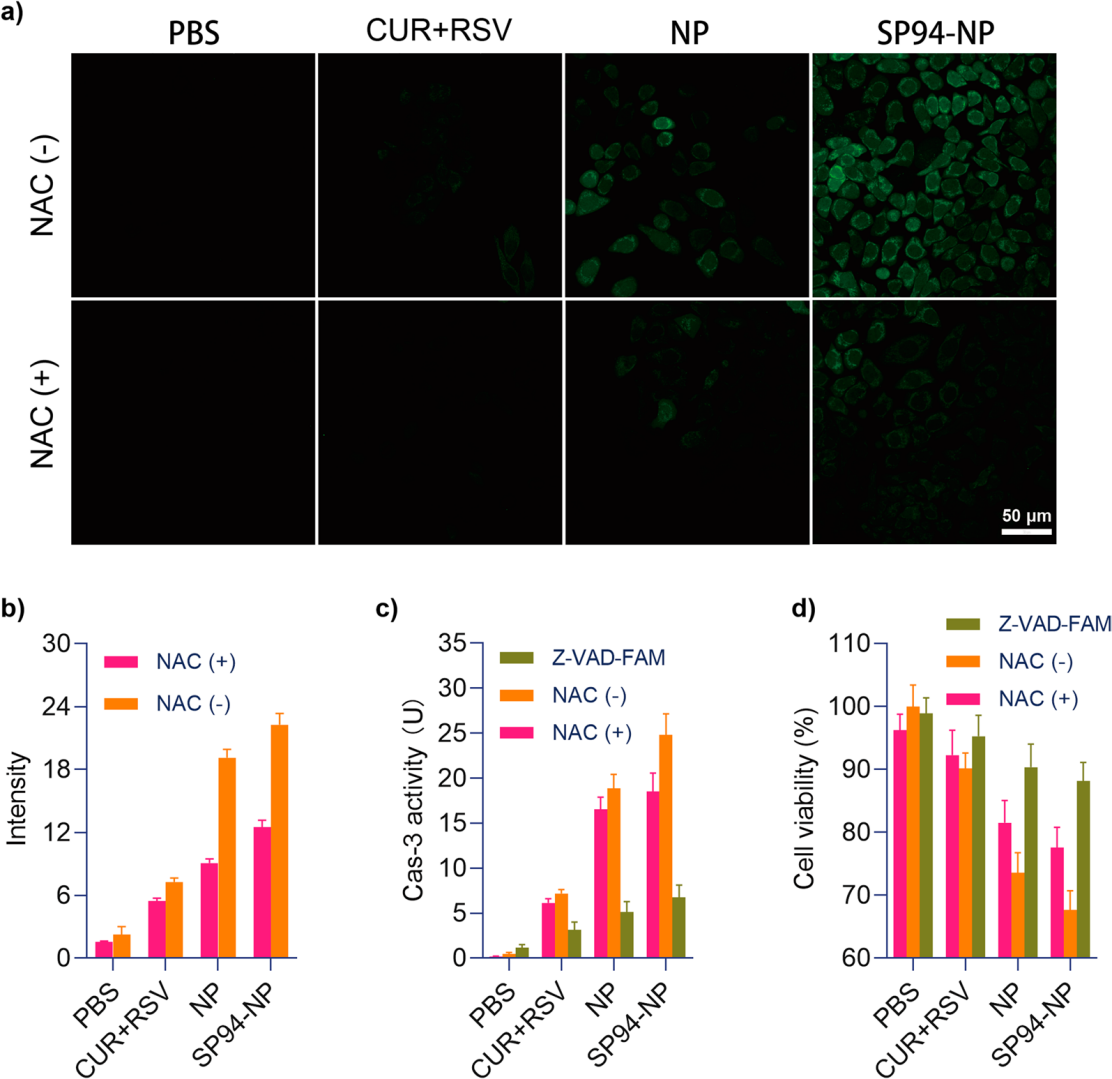
ROS and caspase-3 production induced by NPs. **a**, **b** NPs induced ROS generation in HepG2 cells, which could be inhibited by pretreatment with the ROC inhibitor NAC. The production of intracellular ROS was detected by DCFH-DA staining and observed by inverted-type laser scanning confocal microscopy, and measurements of the average radiant efficiencies. **c**, **d** Caspase-3 activity and cell viability were detected with or without NAC or with the caspase-3 inhibitor Z-VAD-FAM

From a theoretical standpoint, cancer cell apoptosis may result from the NPs inducing the production of ROS and activating a cascade of caspase-3 in HepG2 cells. Excessive ROS can promote cell death, and both CUR and RSV can upregulate ROS-induced apoptosis in cancer cells [48–50]. Therefore, the cell-generated ROS were examined by 2′,7′-dichlorofluorescein diacetate (DCFH-DA) staining. As shown in Fig. 4a, b, HepG2 cells produced significant ROS after NPs treatment, as demonstrated by the observed bright green fluorescence. The apoptosis of cells pretreated with N-acetylcysteine (NAC) was further analysed to elucidate the effects of ROS on apoptosis, and the green fluorescence was greatly reduced. Apart from ROS, apoptosis is carried out by a cascade of caspases (cysteine aspartate-specific proteinases), and activation of caspase-3 is considered a hallmark of apoptosis [51]. The effects of the NPs on the activation of caspase-3 in HepG2 cells were subsequently evaluated. NPs treatment significantly activated caspase-3 in HepG2 cells, with an increasing trend among the four groups. Furthermore, the caspase-3 inhibitor Z-Val-Ala-Asp-CH_2_F (Z-VAD-FMK) completely inhibited NP-induced apoptosis of HepG2 cells (Fig. 4c). It was suggested that the activation of caspase-3 is an important mechanism for the combined effects of the NPs to induce HepG2 cell apoptosis. In addition, NP-induced caspase-3 activity decreased under NAC pretreatment (Fig. 4c). These results indicated that caspase-3 is closely related to the generation of ROS. Moreover, the cell viability of each group after treatment was determined, and the related pathway by which NPs kill HepG2 cells was verified (Fig. 4d). Therefore, we confirmed that these NPs may lead to tumour cell apoptosis by affecting the production of ROS and the activation of caspase-3 in vivo.

### In vivo tumour targeting and NPs distribution

**Fig. 5:**
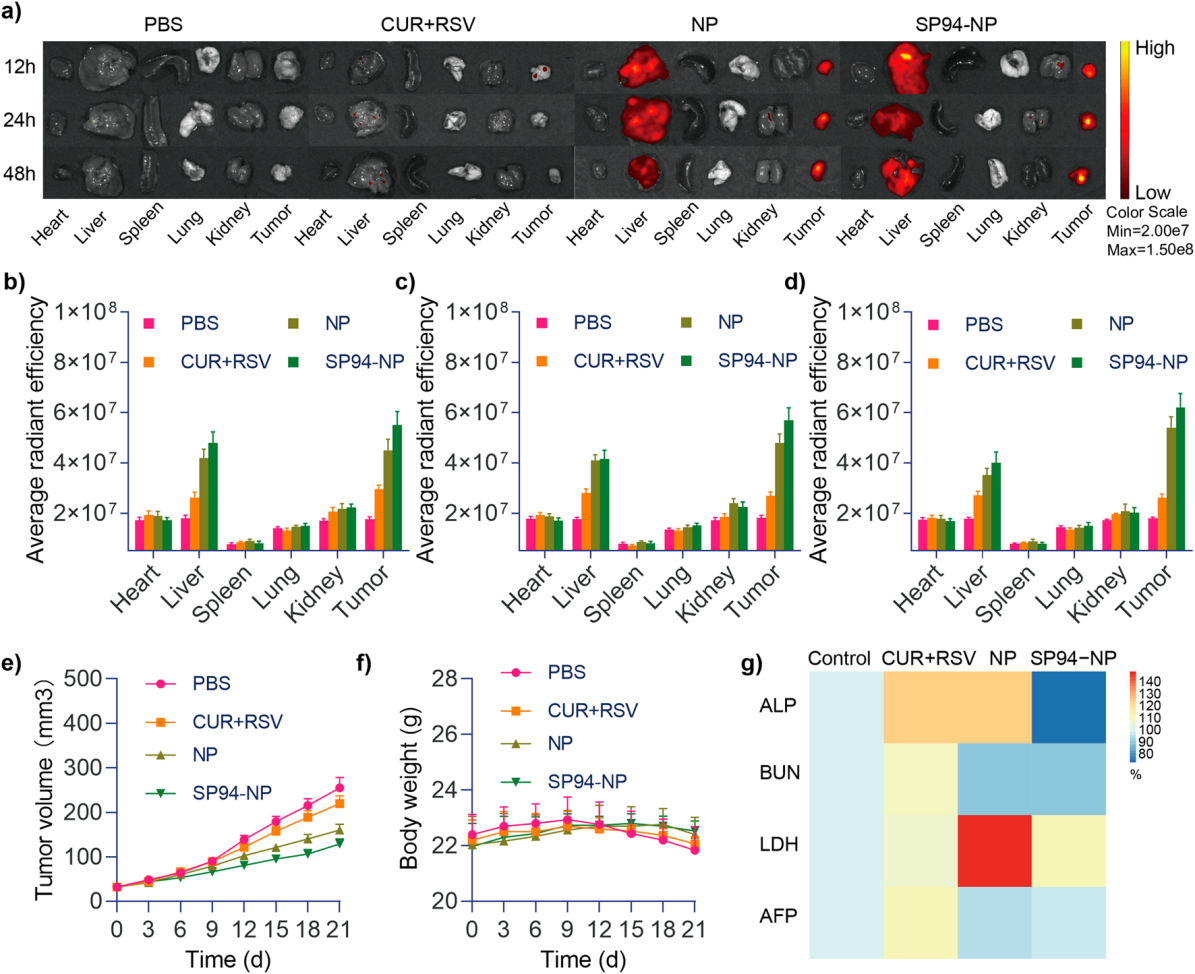
**a** Ex vivo fluorescence imaging. **b**–**d** Quantification of the fluorescence in the major organs and tumours 12, 24 and 48 h after intravenous injection, respectively. **e**, **f** Tumour volumes (mm^3^) and body weights (g) of the HepG2 tumour-bearing mice in the different treatment groups. **g** Routine blood testing results after different treatments

Inspired by the results of the in vitro experiments, we investigated whether modifying the NPs with SP94 would allow preferential drug aggregation in the tumours of BALB/c xenograft-bearing nude model mice. Distribution was observed with an imaging system at 12 h, 24 h and 48 h after injection of solution containing free NPs. As displayed in Fig. 5a–d, the fluorescence signals generated by CUR in the tumour areas of both groups of NP-injected mice tended to converge gradually over time. The liver also displayed some signals because it is the metabolic site of CUR and RSV. The fluorescence signal of free CUR + RSV at the tumour site was much lower than that observed at the tumour site of the mice receiving NPs, and this signal decayed rapidly after the early time points, which indicated that the drugs were quickly metabolized; this finding is in line with the low bioavailability of CUR and RSV. These results clearly demonstrated the tumour-targeting ability of the NPs that was obtained by exploitation of the EPR effect. The active targeting was further conferred and enhanced by modification with the tumour-specific ligand peptide SP94, showing that the NPs are prone to accumulate in the tumour site.

### In vivo evaluation of antitumor activity

**Fig. 6:**
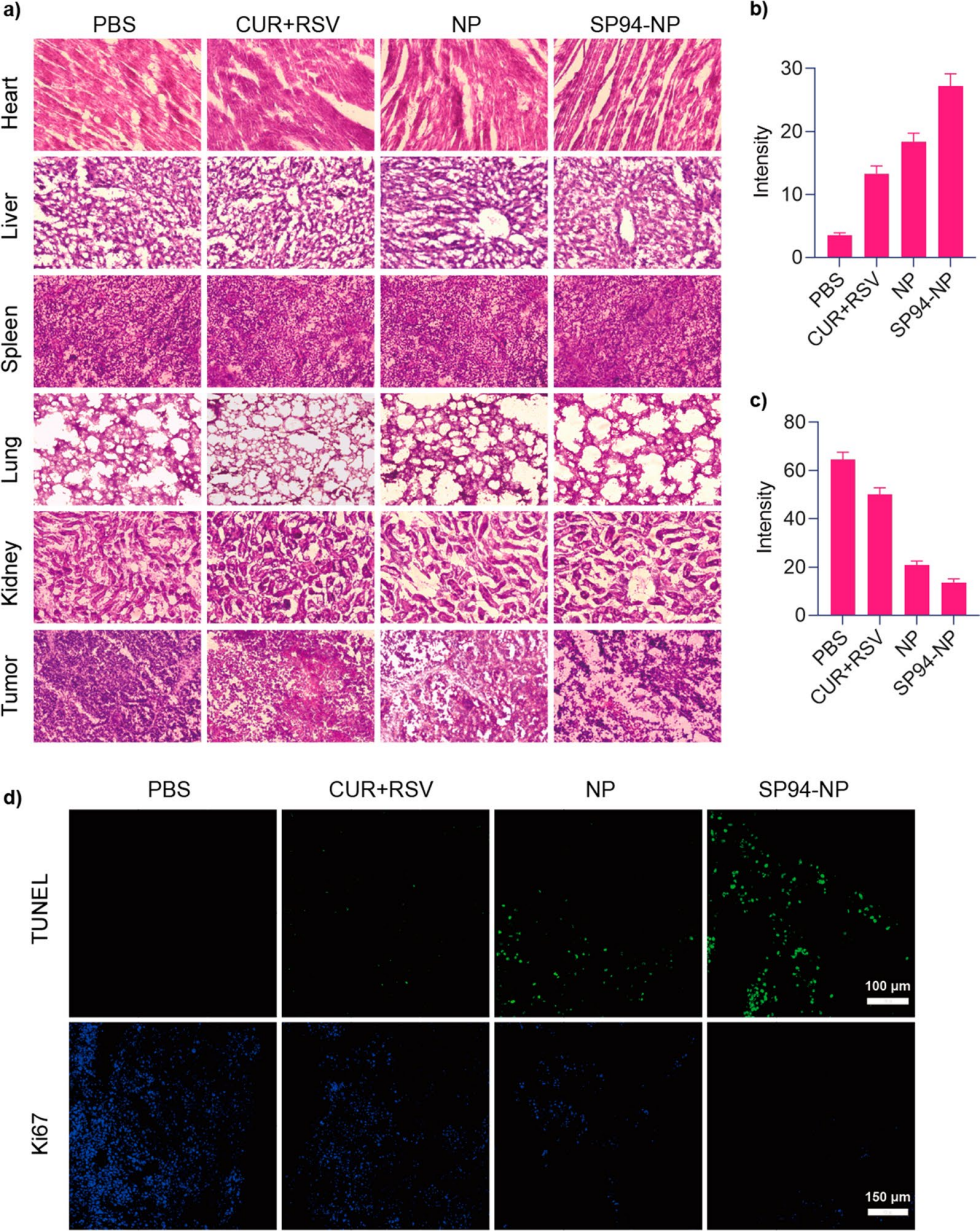
**a** Representative H&E-stained sections of the heart, liver, spleen, lung, kidney and tumour from the tumour-bearing mice from different treatment groups. **b**–**d** TUNEL and Ki-67 staining of tumour sections from different groups of mice after 21 days of treatment and their average radiant efficiencies, respectively

Finally, tumour growth was clearly inhibited in the model mice that underwent NPs treatment (Fig. 5e). In particular, targeted SP94-NP therapy resulted in more significant tumour suppression than nontargeted NP therapy. Conversely, the weights of the mice treated with NPs were remarkably higher (Fig. 5f). On the one hand, this effect may be related to limiting tumour overgrowth, while on the other hand, it illustrated the safety of the NPs. Examinations of blood samples from each group (Fig. 5g) showed that there were no significant changes in ALP, BUN, or LDH, which indicated that the NPs had little effect on liver, kidney and heart function and were highly safe in the mouse model. AFP is the most commonly used marker for liver cancer, and its level was also relatively low in the NP treatment group, which suggested that the NPs were more effective. Haematoxylin and eosin (H&E) staining analysis demonstrated that NPs treatment led to intratumoral necrosis (Fig. 6a), and H&E staining of the heart, liver, spleen, lung, and kidney tissues displayed no obvious abnormalities or organ damage, which further illustrated their efficacy and safety.

Ki-67 and terminal deoxynucleotidyl transferase-mediated dUTP nick end labelling (TUNEL) immunofluorescence assays further confirmed the therapeutic effects of the NPs (Fig. 6b–d). The TUNEL assay revealed gradual increases in the green fluorescence signals of the four groups, which suggested a gradual increase in apoptosis of the tumour cells. Ki-67 detection showed that the fluorescence signals of the four groups gradually diminished, which indicated that tumour proliferation gradually decreased. Thus, the Ki-67 and TUNEL results were consistent and demonstrated that targeted SP94-NPs had significant therapeutic effects in vivo by increasing tumour apoptosis and inhibiting tumour proliferation.

### Conclusions

In conclusion, a new formulation was created to alleviate the disadvantages of low water solubility, weak chemical stability, and poor bioavailability of CUR and RSV while enhancing their therapeutic potential against HCC with a good safety profile. CUR and RSV were rationally designed based on their shortcomings and potential anticancer activities and assembled with the biocompatible matrix DSPE-PEG_2000_. The particle surfaces were decorated with HCC-specific ligands (peptide SP94). The hardening of the liposome membrane caused by CUR and RSV promoted the prolonged residence time of the drugs in the NPs reservoir, thereby hindering their release into systemic circulation. Furthermore, the targeting peptide has high selectivity for cancer cells without affecting normal cells and tissues. Therefore, by properly exploiting the EPR effect and the accumulation of active NPs, the concentration of the therapeutic drugs at tumour target site increased. Finally, as a result of the modular assembly strategy, the individual building blocks can be easily changed. Therefore, it has been speculated that because many traditional chemotherapeutic drugs have substantial side effects, this nanoplatform can be used to minimize these side effects while improving their therapeutic effects.

## Methods

### Materials

1,2-Distearoyl-sn-glycero-3-phosphoethanolamine-N-[maleimide (polyethylene glycol)-2000] (DSPE-PEG_2000_-Mal) was obtained from Avanti Polar Lipids Inc. (Alabaster, AL, USA). Curcumin (CUR), resveratrol (RSV), cholesterol, 1,2-distearoyl-sn-glycero-3-phosphocholine (DSPC) and the peptide SP94 (sequence: CGGSFSIIHTPILPL) were obtained from MeloPEG Biochem Co., Ltd. (Shanghai, China).

### Cell culture and experimental animals

The human hepatoma cell line HepG2 was cultured in DMEM supplemented with 10% foetal bovine serum (FBS) and penicillin (100 IU/mL)/streptomycin (100 μg/mL). All cells were maintained in a 5% CO_2_ humidified incubator at 37 °C. Male 4–5-week-old BALB/c mice were provided by Cavens Laboratory Animal Co. Ltd. (Changzhou, China).

### Synthesis of the SP94-modified lipid DSPE-PEG_2000_ (DSPE-PEG_2000_-SP94)

DSPE-PEG_2000_-SP94 was prepared via a coupling reaction between a maleimide group on DSPE-PEG_2000_-Mal and a cysteine residue in SP94. Briefly, 500 mg of DSPE-PEG_2000_-Mal was dissolved in 3 mL of anhydrous chloroform, and 250 mg of SP94 dissolved in 10 mL of anhydrous dimethyl sulfoxide (DMSO) was added under the protection of nitrogen. Subsequently, 15 μL of triethylamine was added to the reaction solution, which was sealed and stirred for 24 h at room temperature. Analytical high-performance liquid chromatography (HPLC) was adopted to monitor the completion of the reaction. The reaction mixture was then dialyzed against methanol (retained molecular weight: 500 Da) for 48 h and lyophilized for the next experiment [52].

### Preparation of NPs loaded with CUR + RSV

The nanoprecipitation method was adopted to prepare CUR + RSV-loaded polymeric NPs. The NPs were prepared with RSV, CUR, linker lipid (DSPE-PEG_2000_ or DSPE-PEG_2000_-SP94), DSPC and cholesterol at the molecular ratio of 2:0.4:0.6:6:3. In brief, a total weight of 20 mg of RSV, CUR, linker lipid, DSPC and cholesterol was dissolved in 5 mL of chloroform. Afterwards, the chloroform mixture was added to a 25 mL round-bottomed flask, and the solvent was removed by rotary evaporation until a uniformly thin film formed. After hydration, the solution treated with ultrasound for 10 min and extruded through a 200 nm polycarbonate membrane 3 times.

### Particle morphology study by transmission electron microscopy (TEM)

Morphological examination of the NPs was performed by TEM (Tokyo, Japan). Briefly, 10 μL of freshly prepared NPs were added to pure carbon film copper mesh and evaporated and dried at room temperature. Then, 10 μL of 1% uranium acetate solution was added to the copper mesh. After 1 min, the samples were dried with filter paper and placed in a dryer to dry overnight at room temperature before being observed by TEM.

### Particle size and zeta potential

The particle sizes and zeta potentials of the NPs were determined using a Malvern Nano-ZS90 instrument (Malvern, UK) at 25 °C. The changes in particle size over seven days were also measured by the same method.

### Determination of encapsulation efficiency (EE)

EE was measured by UV spectrophotometry. Briefly, RSV and CUR were dissolved in methanol at different concentrations, and concentration curves were generated with measurements from a UV–VIS spectrometer (Shimadzu, UV-2700) [30]. Next, the NPs were dissolved in methanol, and the mixture was stirred at 37 °C for 30 min to demulsify and release CUR and RSV. The total amounts of CUR and RSV released were quantitatively determined based on the concentration curves. Furthermore, NPs were centrifuged at 12,000*g* in a microsep for 10 min, after which the solution was dissolved in methanol, and the amounts of free drugs were measured by the same method. The EE percentages of CUR and RSV in the NPs were calculated via the following equation:

$${\text{EE }}\left( \% \right) = {\text{W}}_{{\text{total drug in NP}}} - {\text{W}}_{{\text{free drug in NP}}} /{\text{W}}_{{\text{total drug in NP}}} \times 100\% .$$

### In vitro release kinetics study and solubility changes

An in vitro release kinetics study was conducted utilizing the dialysis method in phosphate buffered-saline (PBS; pH 7.4) to quantify the drug release behaviour. NPs were loaded into dialysis bags and continuously and gently stirred at 37 °C. Next, 0.5 mL aliquots were collected and replaced with the same volume of fresh PBS at predetermined time intervals. The amounts of CUR and RSV released were measured using a UV–Vis spectrometer. A small animal imaging instrument was used to detect the radiant efficiency of equal masses of CUR and RSV in PBS, NPs and methanol to intuitively judge the changes in solubility. The excitation wavelength was 425 nm, and the emission wavelength was 530 nm.

### Cell-specific uptake of NPs

CUR is fluorescent and can be used to examine the efficiency of NPs uptake by HepG2 cells. Tumour cells were seeded onto 20 mm glass bottom cell culture dishes at a density of 5 × 10^4^ cells for 24 h. Then, the cells were exposed to PBS, CUR + RSV and NPs for 1.5 h followed by washing twice with cold PBS to remove free NPs. Afterwards, the cells were counterstained with SYTO 63 with red fluorescence (5 μL/mL) for 30 min and washed with PBS again. Finally, the cells were observed and photographed using inverted-type laser scanning confocal microscopy (LSCM) (Leica TCS SP8 confocal microscope) with a 100 × oil immersion objective.

### In vitro cell apoptosis studies

HepG2 cells were seeded at 10,000 cells/well and cultured in a 96-well flat bottom plate for 24 h at 37 °C with 5% CO_2_. All cells were treated with RSV, CUR, CUR + RSV (5:1 molar ratio), NP-CUR + RSV and SP94-NP-CUR + RSV (referred to as NP and SP94-NP). The concentrations of CUR and RSV varied from 1 to 500 μM. Then, the plates were incubated for 24 h, and cell viability was assessed using 3-(4,5-dimethylthiazol-2-yl)-2,5-diphenyltetrazolium bromide (MTT). Briefly, 20 μL of MTT solution (5 mg/mL) was added to each well, and the cells were incubated for 4 h according to the manufacturer's instructions. Then, 200 μL of DMSO was used to replace the cell culture medium, and the absorbance (OD) of the DMSO solution was measured with a microplate reader (Bio-Rad Laboratories, Inc., Richmond, California, USA) at 490 nm. Cell viability was calculated by the following formula:

$$\left[ {{\text{OD}}_{{\text{treated cells}}} /{\text{OD}}_{{\text{control cells}}} } \right] \times 100\% .$$

Cell viability data are presented as the mean IC_50_ ± SD for individual drug treatments. For combination treatments, combination index (CI) analysis was conducted using CompuSyn software version 1.00 (Paramus, NJ) [53]. The median-effect principle and the combination index-isologram theorem were adopted to determine synergism or antagonism according to the following equation:

$$\left( {\text{D}} \right)_{1} /\left( {{\text{D}}_{{\text{x}}} } \right)_{1} + \left( {\text{D}} \right)_{2} /\left( {{\text{D}}_{{\text{x}}} } \right)_{2} + \left( {\text{D}} \right)_{3} /\left( {{\text{D}}_{{\text{x}}} } \right)_{3} = {\text{CI}}$$

where D represents the drug and subscripts 1, 2 and 3 refer to the individual drugs in the equation. The subscript x indicates the % inhibition at which a given dose of the drug inhibits cell proliferation. CI values of < 1, 1, and > 1 indicate synergism, additivity, and antagonism, respectively. The CI vs. fractions of cells affected (fa) provides the interaction at all effect levels (1–99%) for a given combination. Moreover, the optimal incubation time with drugs was evaluated at 0.5, 1.5 and 2.5 h.

### Detection of reactive oxygen species (ROS) and caspase-3 activity

2′,7′-Dichlorofluorescein diacetate (DCFH-DA) staining was used to detect intracellular ROS production. HepG2 cells (5 × 10^4^) were placed in 20 mm culture dishes for 24 h. Then, the cells were exposed to PBS, CUR + RSV and NPs for 1.5 h and stained with DCFH-DA (10 M) at 37 °C for 20 min. DCF fluorescence was quantified with a confocal microscope at 488^ex^ nm/525^em^ nm [54]. For ROS inhibition, all cells were pretreated with N-acetylcysteine (NAC; ROS scavenger) (2.5 mmol for 2 h), followed by drug treatment [55]. To evaluate the effects of the NPs on caspase-3, caspase-3 activity was determined by measuring the absorbance of specific chromogenic substrates according to the manufacturer’s instructions. Moreover, in another group, the cell-permeable pan-caspase inhibitor Z-Val-Ala-Asp-CH_2_F (Z-VAD-FMK) (20 μmol for 30 min) was used to inhibit the production of caspase-3 before drug treatment [56]. Finally, cell viability in these groups was measured according to the MTT method.

### Antitumor efficacy evaluation in vivo

Four-week-old male BALB/c nude mice were subcutaneously implanted with HepG2 cells (1 × 10^7^ cells/150 μL/animal) to evaluate the antitumor effects of our NPs in a human-derived HCC tumour model. After the tumour sizes grew to be visible, all mice were randomly divided into four groups: PBS, CUR + RSV, NP and SP94-NP. The mice were gavaged with 10 mg/kg CUR and 50 mg/kg RSV (with equivalent doses in the NP groups) every 3 days through the tail vein. The treatment lasted 21 days, and tumour volumes and body weights were recorded regularly. Tumour volume was calculated using the following formula: tumour volume (mm^3^) = 0.5 × length × width^2^. The mice were sacrificed on Day 21 posttreatment, and the frozen tumour tissue sections were incubated with a terminal deoxynucleotidyl transferase-mediated dUTP nick end labelling (TUNEL) reaction mixture (Roche Diagnostics) at 37 °C for 1 h. The slides were then visualized under a fluorescence microscope [57]. Additionally, routine Ki-67 immunohistochemistry was carried out according to the manufacturer’s instructions and visualized under a fluorescence microscope [57].

### Toxicity and biosafety of the NPs in vivo

Blood samples were obtained on Day 21 to detect the ALP, BUN, LDH and AFP levels based on the manufacturer’s instructions. Briefly, these indicators were determined by setting blank, control and experimental groups, and their OD values were detected. Then, their corresponding concentrations were calculated according to the formula:

$${\text{Concentration}}\, =\, ({\text{OD}}_{{{\text{experiment}}}} - {\text{OD}}_{{{\text{blank}})}} /\left( {{\text{OD}}_{{{\text{control}}}} - {\text{OD}}_{{{\text{blank}}}} } \right) \times {\text{standard concentration}} \times {\text{diluted multiples}}.$$

The tumour tissues and various organs were collected, dissected, fixed with paraformaldehyde (4%), and subjected to haematoxylin and eosin (H&E; Sigma) staining for histological analysis.

### In vivo tumour targeting and NPs distribution

Twelve, 24 and 48 h after drug administration via the tail vein, the mice were sacrificed. The hearts, livers, spleens, lungs, kidneys and tumours of the xenograft model mice were collected, and a Calliper IVIS Lumina II in vivo imaging system (Calliper Life Science, USA) was used to detect the time-dependent drug distribution in the tumour tissues and other major organs.

### Statistical analysis

All quantitative data are expressed as the mean ± SD. Statistical analysis of the experimental data was performed using GraphPad Prism V8.0 (GraphPad Software, USA), and statistical significance was defined as a p < 0.05 from two-sided tests.

## Supplementary Information

**Additional file 1: Figure S1.** The mass spectrum (MS) of SP94 peptide. **Figure S2.** The purity analysis by HPLC for SP94 peptide. **Figure S3.** HPLC report of (a) SP94 (0.4 mg/mL) and (b) SP94-DSPE-PEG(2000) (0.4 mg/mL). **Figure S4.** ^1^H NMR characterization of the SP94-DSPE-PEG(2000). The ^1^H NMR spectrum was measured in deuterated DMSO.

## Data Availability

All data generated or analyzed during this study are included in this published article.
