# Expression of Concern: Curcumin- and resveratrol-co-loaded nanoparticles in synergistic treatment of hepatocellular carcinoma

**DOI:** 10.1186/s12951-025-03361-7

**Published:** 2025-04-10

**Authors:** Yongshun Zheng, Ran Jia, Jun Li, Xiaohe Tian, Yeben Qian

**Affiliations:** 1 Department of General Surgery, The First Affiliated Hospital of Anhui Medical University, 218 Jixi Road, Hefei, 230022 Anhui China; 2 Department of Radiology and National Clinical Research Center for Geriatrics, Functional and Molecular Imaging Key Laboratory of Sichuan Province, Huaxi MR Research Centre (HMRRC), West China Hospital of Sichuan University, Chengdu, 610000 China; 3 Department of Chemistry, Key Laboratory of Functional Inorganic Material Chemistry of Anhui Province, School of Life Science, Anhui University, Hefei, 230000 China

**Expression of Concern: Journal of Nanobiotechnology (2022) 20: 339**

10.1186/s12951-022-01554-y

The Editor-in-Chief would like to alert the readers that concerns have been raised regarding the data presented in Fig. 6a.

After publication, a reader noted high similarity between Fig. 6a Spleen images representing the CUR + RSV and NP groups. The authors stated that the images from the same sample were included in the figure by mistake, and provided the full original data for validation. However, further checks by the Publisher and Associate Editors identified potential issues in sample preparation in the majority of the original images, deeming that the data were insufficient to fully support the authors’ statement that the tissues displayed no abnormalities or organ damage.

Readers are therefore advised to interpret these results with caution.

Yeben Qian has stated on behalf of all authors that they agree to this Editorial Expression of Concern.

