# Supplementary material for: Curcumin- and resveratrol-co-loaded nanoparticles in synergistic treatment of hepatocellular carcinoma
Source: J Nanobiotechnology. 2022 Jul 20;20:339. doi: 10.1186/s12951-022-01554-y (PMC9301856; doi:10.1186/s12951-022-01554-y)
Supplement: Supplementary file 1 — Additional file 1: Figure S1. The mass spectrum (MS) of SP94 peptide. Figure S2. The purity analysis by HPLC for SP94 peptide. Figure S3. HPLC report of (a) SP94 (0.4 mg/mL) and (b) SP94-DSPE-PEG(2000) (0.4 mg/mL). Figure S4. 1H NMR characterization of the SP94-DSPE-PEG(2000). The 1H NMR spectrum was measured in deuterated DMSO. [file 12951_2022_1554_MOESM1_ESM.docx]

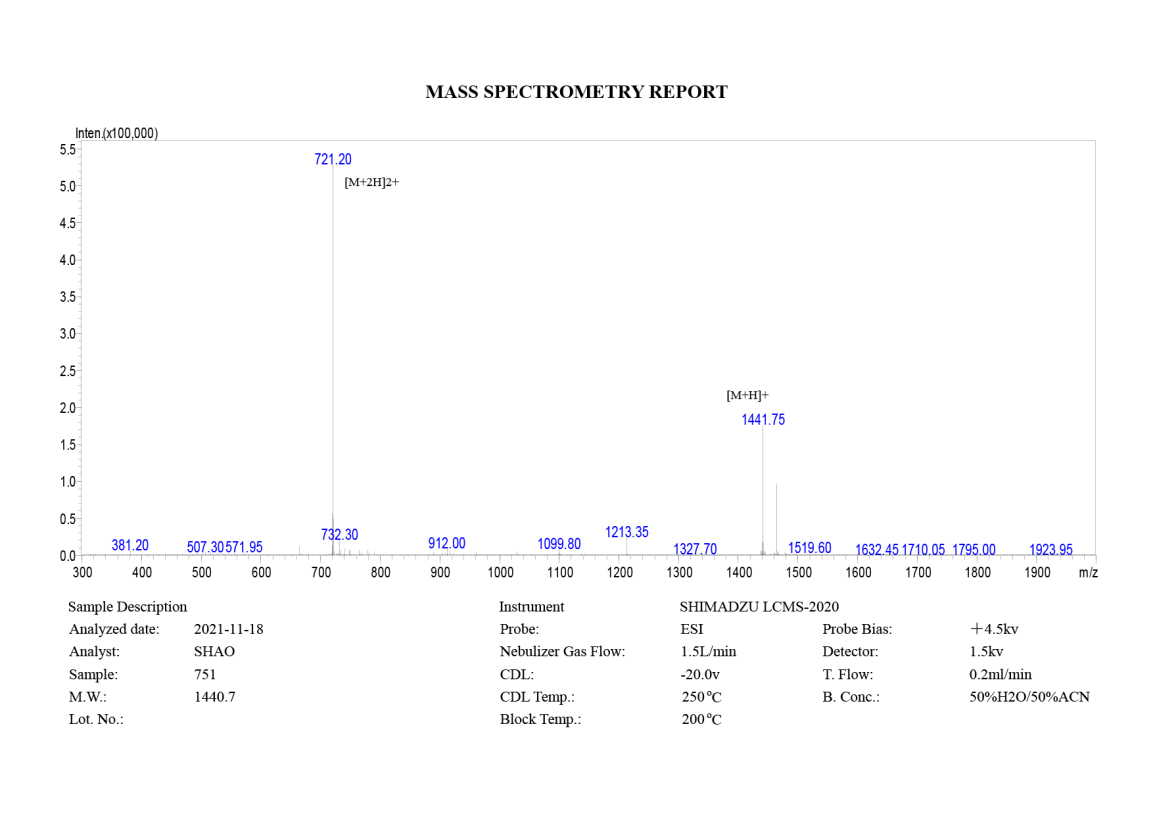


Figure S1. The mass spectrum (MS) of SP94 peptide.


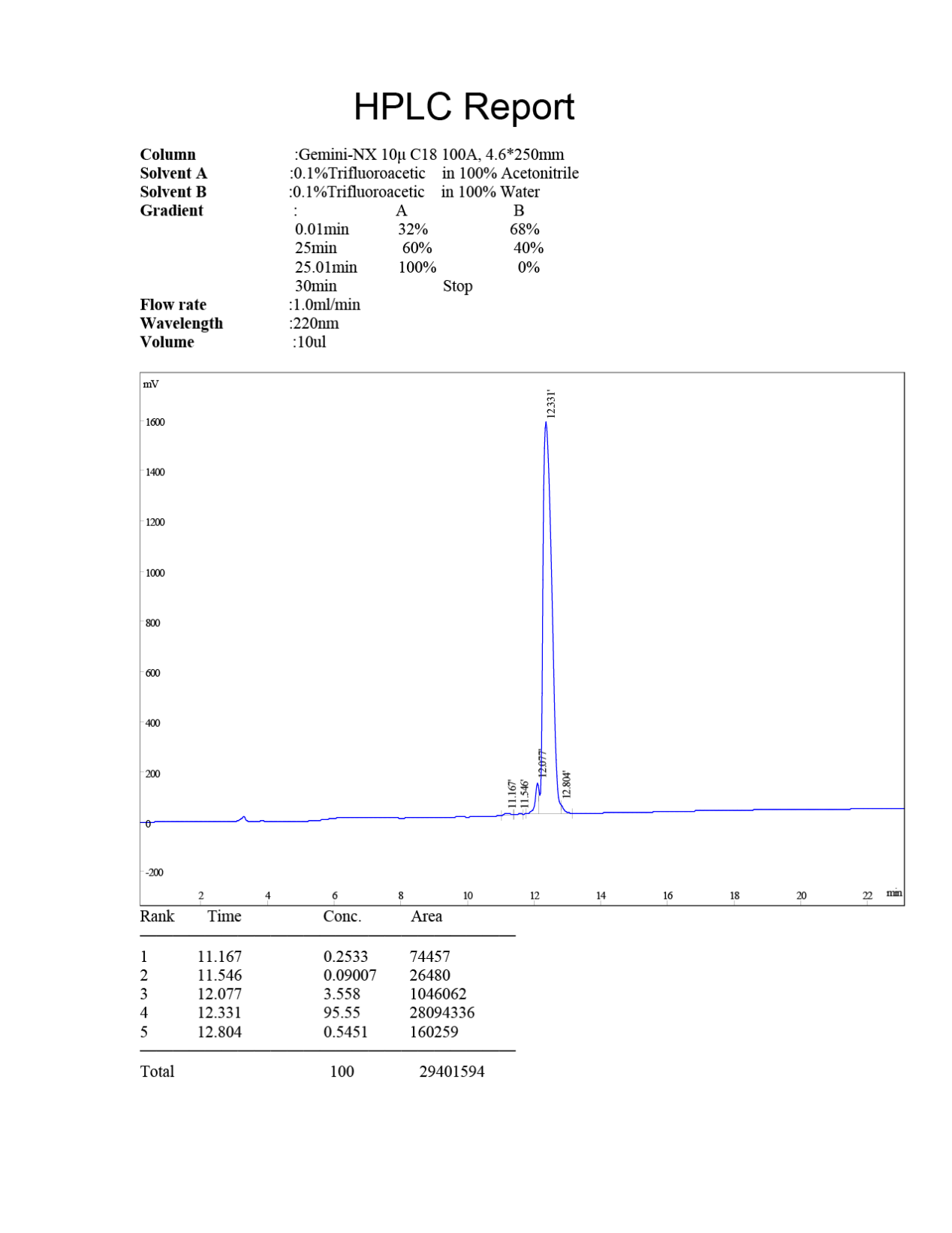


Figure S2. The purity analysis by HPLC for SP94 peptide.


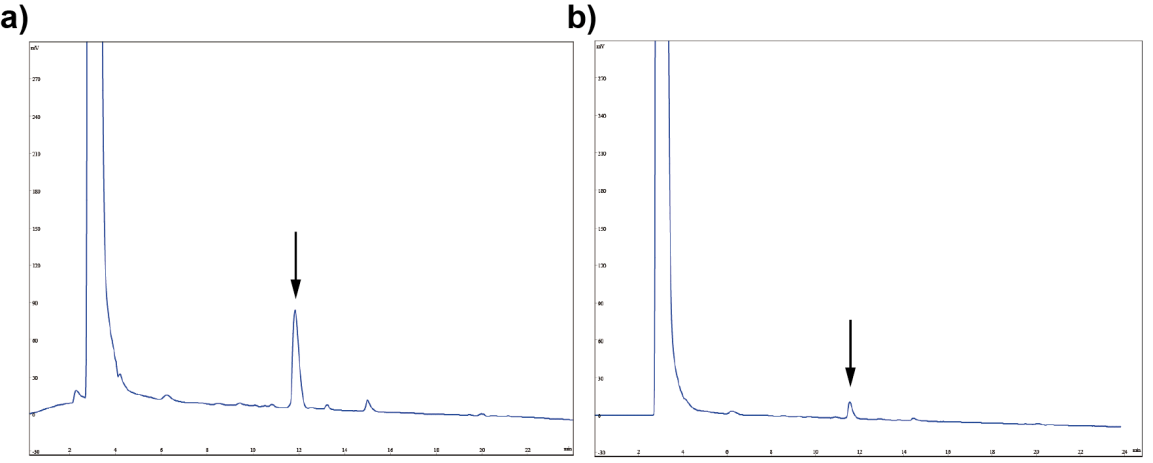


Figure S3. HPLC report of (a) SP94 (0.4 mg/mL) and (b) SP94-DSPE-PEG(2000) (0.4 mg/mL). The arrows show the peaks corresponding to SP94 and SP94-DSPE-PEG(2000), respectively. The HPLC conditions are:

Column :Gemini-NX 10μ C18 100A, 4.6*250mm

Solvent A :0.1%Trifluoroacetic in 100% Acetonitrile

Solvent B :0.1%Trifluoroacetic in 100% Water

Gradient : A B

0.01min 30% 70%

25min 55% 45%

25.01min 100% 0%

30min Stop

Flow rate :1.0ml/min

Wavelength :220nm

Volume :10ul


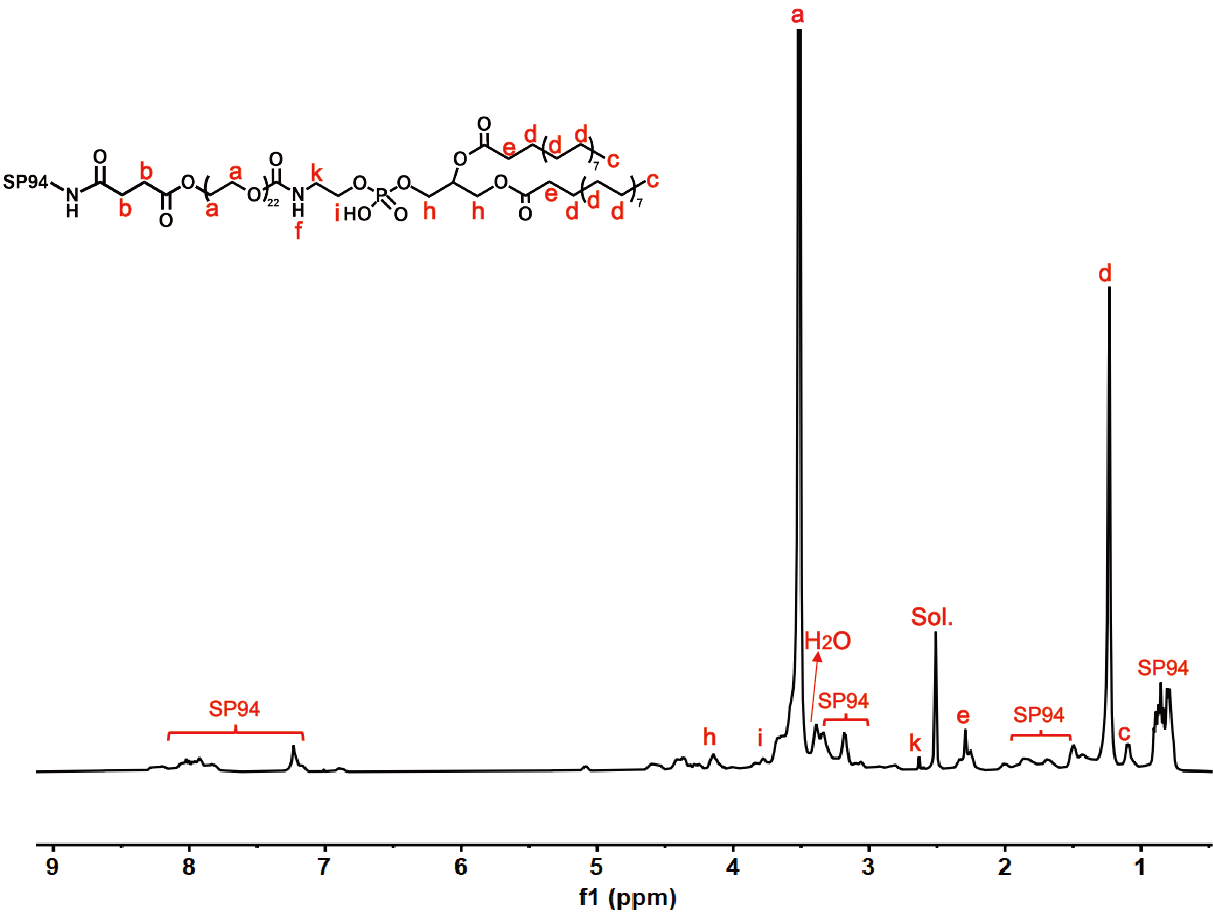


Figure S4. ^1^H NMR characterization of the SP94-DSPE-PEG(2000). The ^1^H NMR spectrum was measured in deuterated DMSO.
